# Extinction Risk Assessment and Conservation of the *Pachypodium* Under Climate Change

**DOI:** 10.1002/ece3.71926

**Published:** 2025-08-08

**Authors:** Yu Chen, Qisong Wan, Shenglan Du, Hillary Otieno Otieno, Haingotiana Johary Andrianjatovo, Maxwell Njoroge Njenga, Yuvenalis Morara Mbuni, Neng Wei, Jitao Li, Shengwei Wang

**Affiliations:** ^1^ School of Forest and Horticulture Hubei Minzu University Enshi China; ^2^ State Key Laboratory of Plant Diversity and Specialty Crops, Wuhan Botanical Garden Chinese Academy of Sciences Wuhan China; ^3^ School of Ecology and Environment Tibet University Lhasa China; ^4^ University of Chinese Academy of Sciences Beijing China; ^5^ Department of Plant Biology and Ecology, Faculty of Sciences University of Antananarivo Antananarivo Madagascar; ^6^ East African Herbarium National Museums of Kenya Nairobi Kenya; ^7^ Sino‐Africa Joint Research Center Chinese Academy of Sciences Wuhan China

**Keywords:** climate change, endemism, habitat suitability, IUCN, machine learning, *Pachypodium*

## Abstract

Global climate change poses unprecedented challenges to the maintenance and survival of biodiversity, with endemic species in particular regions facing an exceptionally high risk of extinction. *Pachypodium*, a genus endemic to South Africa and Madagascar, exhibits strong habitat specificity, yet the impacts of climate change on its distribution patterns remain not fully understood. This study employs the Biomod2 package in R to predict changes in the distribution patterns of 20 *Pachypodium* species under climate change scenarios (SSP2‐4.5 and SSP5‐8.5). Additionally, machine learning methods have been applied to assess the extinction risk of these species. The results indicate that climate change will severely impact the distribution of the genus *Pachypodium*. Suitable habitat areas for 15 species within the genus are projected to shrink significantly in the future, with the most pronounced habitat loss occurring in central and eastern Madagascar, eastern Namibia, and central and northern South Africa. Annual precipitation and precipitation seasonality are the main factors influencing these habitat changes. A reassessment of the IUCN categories for *Pachypodium* reveals that the number of threatened species will increase from 7 to 13. Alarmingly, three species are predicted to face a risk of extinction in the wild due to climate change. Moreover, the current protected areas have proven ineffective in safeguarding the habitats of *Pachypodium*, with protected habitats expected to decrease by 30.39% under the influence of climate change. These findings provide strategic insights for the conservation of *Pachypodium* species and highlight the necessity for reforms and adaptive adjustments to current protected area networks to address the challenges posed by climate change.

## Graphical abstract

Climate change impacts on *Pachypodium*: Future climate scenarios predict a significant reduction in suitable habitats, with severe losses in Madagascar and southern Africa. Increased threat levels: The reassessment of *Pachypodium* under IUCN criteria shows the number of threatened species rising from 7 to 13, with three species facing potential extinction in the wild. Urgent need for adaptive conservation: The current protected areas have failed to effectively safeguard *Pachypodium* habitats and are even less capable of addressing the impacts of climate change.

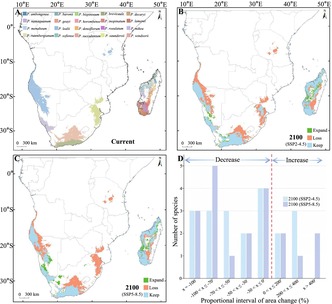

## 1 Introduction

The Earth is currently undergoing a biodiversity crisis, with species extinction rates far exceeding the pace of natural evolution (Barnosky et al. 2011; Cardillo et al. 2023; Thuiller et al. 2005). While natural climate fluctuations and nonclimatic factors contribute to these trends, climate change is widely regarded as the most significant threat to biodiversity in the 21st century (Urban 2015). Recent studies show that a global temperature increase of 1.3°C would put 1.6% of species at risk, whereas a rise of 5.4°C could increase this risk to 29.7% (Urban 2024). Plants, as foundational components of ecosystems, play a critical role in maintaining ecological balance and supporting human well‐being. However, compared with animals, plants often receive inadequate attention in conservation initiatives (Brummitt et al. 2015). With the ongoing intensification of the sixth mass extinction event, over 20% of plant species are now at risk of extinction, and many are disappearing before they can even be scientifically documented (Borgelt et al. 2022; Westwood et al. 2021). To address this escalating biodiversity crisis, the Convention on Biological Diversity (CBD) adopted the Kunming‐Montreal Global Biodiversity Framework (KMGBF), which outlines strategic measures to combat biodiversity loss (www.cbd.int). The framework emphasizes the intricate linkage between climate change and biodiversity and advocates for global collaboration to implement effective conservation strategies aimed at preserving and restoring ecosystem diversity (McGowan et al. 2024; Williams et al. 2020). Climate change undermines the stability of global plant diversity by reshaping species distributions and altering habitat suitability (Tilman et al. 2017; Zhang et al. 2024). Biodiversity hotspots regions, which harbor exceptional concentrations of species diversity and endemism, are particularly vulnerable to the impacts of climate change (Trew and Maclean 2021).

Africa is home to some of the world's most prominent biodiversity hotspots (Habel et al. 2019; Hopper et al. 2015), and harbors an exceptionally high level of plant endemism (Gallagher et al. 2023). The continent hosts over 65,000 vascular plant species (Qian et al. 2021), with many regions exhibiting a high proportion of endemic species, particularly in biodiversity hotspots such as Madagascar and the Cape Floristic Region in South Africa (Gallagher et al. 2023). However, climate change has significantly impacted various regions of Africa, including delayed rainy seasons in West Africa, more frequent extreme rainfall events in southern Africa, and a declining trend in precipitation in Madagascar (Fotso‐Nguemo et al. 2022; Randriamarolaza et al. 2022). These shifts in climatic patterns are altering habitat conditions, posing increasing survival challenges for Africa's endemic plants. For instance, certain legume species endemic to the Congo region have exhibited a high degree of sensitivity to projected climate change scenarios, with their climatically suitable habitats undergoing a progressive contraction (Oyebanji et al. 2021). This vulnerability stems from their restricted geographic ranges and strong habitat specificity, which make them particularly susceptible to environmental changes, often resulting in significant habitat loss and range contraction (Cogoni et al. 2021; Kidane et al. 2019). For instance, under climate change, the native habitats of the East African endemics *Aloe classenii* and *A. ballyi* have been destroyed by over 34% and 44%, respectively (Mkala et al. 2022); under the most pessimistic climate change scenario (RCP8.5), the suitable habitat area for *Clivia miniata* in South Africa is projected to decrease by 14.2% ± 9.6% by 2050 (Groner et al. 2022). Meanwhile, *Adansonia rubrostipa* and *A*. *za*, two baobab species from Madagascar, are facing severe habitat loss and fragmentation, resulting in the destruction and disappearance of large areas of their original habitats (Wan et al. 2021). Many endemic plants have been classified as endangered by the International Union for Conservation of Nature (IUCN, www.iucnredlist.org), which has significantly increased public awareness of their conservation status and supported protective efforts. For instance, sustained scientific focus and a suite of conservation‐driven research initiatives targeting Madagascar's six endemic *Adansonia* species (IUCN‐assessed) have generated demonstrable conservation benefits (Wan et al. 2021; Wan et al. 2024). However, endemic plants that are still not assessed by the IUCN may be overlooked or underestimated and may face extinction risks, particularly with regard to their vulnerability to climate change (Holz et al. 2022; Nic Lughadha et al. 2020). For example, in Madagascar, only one‐third of plant species have been formally assessed by the IUCN, a critical gap underscoring the need for additional resources and technical capacity to enhance species assessment efforts (Ralimanana et al. 2022).

The genus *Pachypodium* (Apocynaceae), commonly referred to as the bottle tree, is endemic to Madagascar and Southern Africa and comprises 23 species (Agrawal et al. 2018; Burge et al. 2013). Renowned for their diverse growth forms and striking entomophilous flowers, these plants possess significant ecological and scientific research value (Burge et al. 2013; Rapanarivo 1999). All *Pachypodium* species are succulent shrubs or small trees with thickened stems, thriving in arid and semiarid regions, and are recognized for their distinctive morphological traits and ecological adaptations to drought environments. Conservation research on this genus currently remains limited, with only a handful of species receiving significant attention (Guo et al. 2019). Even in the IUCN, only 10 species of *Pachypodium* have been included in the Red List. Of these, *P. inopinatum* is classified as Critically Endangered (CR), *P. baronii* and *P. mikea* as Endangered (EN), *P. brevicaule* , *P. lealii* , and *P. sofiense* as Vulnerable (VU), and *P. geayi* , *P. lamerei* , *P. namaquanum* , and *P. rutenbergianum* as Least Concern (LC). However, the extinction risks posed to *Pachypodium* species by climate change may be underestimated in current IUCN assessments. This is because their arid and semi‐arid habitats are highly susceptible to climate change impacts, including rising temperatures, altered precipitation patterns, and an increased frequency of extreme weather conditions such as droughts and heavy rainfall (Li et al. 2019; Zhou et al. 2024). Furthermore, *Pachypodium* species have been extensively overharvested for horticultural purposes, resulting in a sharp decline in wild populations and severe habitat degradation, further exacerbating their survival challenges (Burge et al. 2013). Therefore, understanding habitat changes in *Pachypodium* species under climate change, assessing their extinction risks, and identifying gaps in future conservation strategies are crucial for safeguarding these plants. Such knowledge also offers valuable insights for the global conservation of plant species threatened by climate change.

Species distribution models play a crucial role in understanding how species respond to climate change and shifts in their distribution patterns (Dakhil et al. 2024; Hosseini et al. 2024). These models integrate species occurrence records with environmental variables to predict the spatial probability of species presence and habitat suitability (Abebe et al. 2024), providing a scientific basis for developing conservation strategies in the context of climate change, such as identifying suitable areas for in situ or ex situ conservation (Vaissi and Mohammadi 2024). Currently, various species distribution models are available for predicting species distributions (Rathore and Sharma 2023). Some commonly used models include artificial neural networks (ANN), classification tree analysis (CTA), generalized additive models (GAM), generalized boosted models (GBM), generalized linear models (GLM), MaxEnt, and random forest (RF) (Liu et al. 2024). Notably, compared with individual models, ensemble models generally exhibit higher accuracy in predicting potential species distributions (Huang et al. 2023).

In this study, we utilized the BCC‐CSM2‐MR global climate model in combination with ensemble modeling from the R package Biomod2 to Predicted changes in suitable habitats and extinction risks faced by *Pachypodium* species under future climate scenarios (SSP2‐4.5 and SSP5‐8.5). Furthermore, by incorporating IUCN Categories and relevant environmental drivers, we applied machine learning techniques to conduct rapid assessments of the conservation status for species that have not been evaluated by the IUCN. Through an analyzing of the overlap between projected future habitats and existing protected areas, key regions will be identified for future conservation planning. The results of this study will provide a scientific basis for effective biodiversity conservation, offer fundamental insights into the adaptive capacity of *Pachypodium* species under climate change, and support the development of appropriate conservation measures for their long‐term protection.

## 2 Materials and Methods

### 2.1 Study Area

**FIGURE 1:**
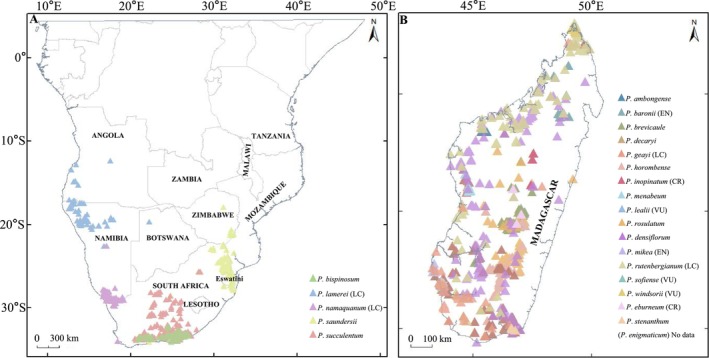
Occurrence records and IUCN categories of the *Pachypodium* species. (A) Five endemic specie in Africa continent. (B) 18 endemic species in Madagascar. Different colors represent different species. CR, critically endangered; EN, endangered; LC, least concern; No data, no record of occurrence; VU, vulnerable. The IUCN categories get form IUCN (https://www.iucnredlist.org/, accessed June 1, 2024).

In this study, we focused on the distribution of *Pachypodium* species in Africa, which located between 11° S–35° S and 12° E–50° E. Madagascar exhibited distinctive climatic and topographic features: its north–south‐oriented eastern mountain range (elevation > 800 m) creates a pronounced precipitation gradient, shaping diverse climatic zones—from humid eastern forests to subhumid central highlands, arid western grasslands, and semidesert southwestern regions (Antonelli et al. 2022). These heterogeneous niches have made Madagascar the center of *Pachypodium* species diversity, with 18 endemic species. In contrast, southern Africa (encompassing South Africa, southwestern Angola, along with northwestern and southern Namibia) is climatically more homogeneous, dominated by arid conditions, and supports only five endemic species (Figure 1A,B) (Cowling et al. 1998).

### 2.2 Species Occurrence Data

Occurrence data for *Pachypodium* species were sourced from online databases. The database consulted include, the Global Biodiversity Information Facility (GBIF.org 2019, https://doi.org/10.15468/dl.jed6dt), the RAINBIO mega database (Rainbio, http://rainbio.cesab.org), and the Tsimbazaza Botanical and Zoological Park Herbarium (TAN, accessed August 27, 2024). In total, 3028 *Pachypodium* records were obtained, with 2969 records sourced from the GBIF database, 3 from the RAINBIO mega database, and 56 from TAN. After data cleaning—removing duplicates, excluding records outside the study area, and omitting those from urban regions—2074 records were retained for subsequent simulation. Considering that insufficient occurrence data could lead to inaccuracies in species distribution models and extinction risk predictions, only 20 species with occurrence data greater than 5 were selected for analysis and research in this study. *P. eburneum* , *P*. *stenanthum*, and *P*. *enigmaticum* were not included in this study (Figure 1; Table S1).

### 2.3 Environmental Variables

Climate data were obtained from WorldClim database (www.worldclim.org), encompassing historical (1970–2000) and future climate data (2081–2100) at a resolution of 2.5 arc minutes. The historical climate data were used to represent current climate conditions. Future climate projections were based on the BCC‐CSM2‐MR climate model (Beijing Climate Center, China Meteorological Administration), a CMIP6‐endorsed model widely recognized for its strong performance in simulating climate patterns, particularly in topographically complex regions. Given its demonstrated accuracy in regional‐to‐global ecological modeling (Rawat et al. 2022; Tan et al. 2024), we selected this model to ensure high reliability in our projections. Two representative CO_2_ emission scenarios were employed: SSP2‐4.5 (low‐emission scenario) and SSP5‐8.5 (high‐emission scenario). Elevation data were sourced from the GMRT database (www.gmrt.org) (Table S2) and resampled to match the resolution of the climate data to ensure consistency across all environmental layers. Pearson correlation coefficients were calculated to prevent model overfitting (Ke et al. 2024), and variables with correlation coefficients exceeding 0.75 were excluded (Figure S1).

### 2.4 Species Distribution Models

The Biomod2 package in R software was utilized to predict the distribution of *Pachypodium* species, integrating five commonly used algorithms (CTA, GBM, GLM, MAXENT, and RF) (Huang et al. 2023; Thuiller et al. 2016). Pseudo‐absence points were randomly generated using the distribution data for each species. Initially, single‐model simulations were performed with each model run 20 times. To validate model reliability, the dataset was divided into 70% for training and 30% for testing, with 20‐fold crossvalidation. The models were evaluated using the Area Under the ROC Curve (AUC) and the True Skill Statistic (TSS), both metrics ranging from 0 to 1. Higher AUC and TSS values indicate superior predictive performance (Liu and Chen 2024). Models with AUC > 0.7 and TSS > 0.7 were considered high‐performing models (Li et al. 2024). These high‐performing models were subsequently integrated using four ensemble methods: mean (EMmean), median (EMmedian), committee averaging (EMca), and weighted mean (EMwmean). The ensemble models were further evaluated using AUC and TSS, and the optimal combination model was selected to simulate suitable habitats for the species (Hao et al. 2020). The natural breaks (Jenks) classification method was used in ArcGIS to calculate the potential suitable habitats area (km^2^) with the Jenks scores ≥ 0.6.

### 2.5 Extinction Risk Assessment of *Pachypodium* Species

#### 2.5.1 Filling the Gaps in the IUCN Categories

Machine learning methods were employed in this study to predict the IUCN categories of species without prior assessments. Specifically, the existing IUCN categories of *Pachypodium* species were used as training data to develop models that predict the IUCN status of unassessed species. The “ConR” and “IUCNN” R packages were used for machine learning and category prediction. The “ConR” package requires the provision of species lists and occurrence records to automatically and batch‐wise assess species IUCN categories and visualize the results. According to the IUCN Standard B, it calculates three core indicators: Extent of Occurrence (EOO), Area of Occupancy (AOO), and the number of subpopulation locations (Cosiaux et al. 2018; Dauby et al. 2017; Stévart et al. 2019). The “IUCNN” package predicts the IUCN categories of species through deep learning techniques. Initially, data on species with known IUCN statuses are prepared as features, while unassessed species serve as labels. During the training process, the model incorporates 37 environmental factors (such as geography, climate, biomes, and human disturbance) to learn patterns of endangered statuses within the same family and genus. It then assigns the most likely IUCN status to each species (Zizka, Andermann, et al. 2021; Zizka, Silvestro, et al. 2021). Both methods support batch assessments but emphasize different aspects of conservation evaluation. To ensure consistency, the higher extinction risk predicted by either method was adopted as the final conservation status for each *Pachypodium* species.

#### 2.5.2 Impact of Climate Change on the IUCN Category of *Pachypodium* Species

Changes in IUCN categories based on climate changes were evaluated by the shifts in potential distribution ranges. According to IUCN Red List Criterion A1(c) (IUCN 2024), species are classified as Extinct in the Wild (EX) when their potential distribution lost is 100%. For CR species, a loss exceeding 90% also results in classification as EX, while other cases retain their current categories. EN species are classified as CR if their potential distribution loss exceeds 90%, and remain as EN for losses between 90% and 70%; other cases retain their existing classification. VU species are classified as CR if the loss exceeds 90%, EN for losses between 90% and 70%, and near threatened (NT) for losses between 70% and 50%, while other cases remain unchanged. For NT species, they are classified as CR for losses exceeding 90%, EN for losses between 90% and 70%, and VU for losses between 70% and 50%, while other cases maintain their existing classification. LC species are classified as EN for losses exceeding 90%, VU for losses between 90% and 70%, and NT for losses between 70% and 50%; other cases maintain their existing classification (Table S3).

For species with expanding distribution ranges, CR species are reclassified as EN for range expansions of 100%–150%, and VU for expansions exceeding 150%. EN species are classified as VU for range expansions of 100%–150%, and NT for expansions exceeding 150%. VU species are reclassified as NT for range expansions of 100%–150%, and LC for expansions exceeding 150%. NT species are reclassified as LC for range expansions exceeding 100% (Table S3).

### 2.6 Conservation Status of *Pachypodium* Species

To understand the distribution of *Pachypodium* under current and future scenarios, we overlaid the current protected area network with potential distribution ranges for different time periods. The proportion of each species' distribution range within protected areas (Inside PAs) and outside protected areas (Outside PAs) was then calculated. Protected area data were obtained from the World Database on Protected Areas (WDPA) dataset (https://www.protectedplanet.net/en).

All calculations and analyses were conducted using R (version 4.2–3) (R Core Team 2023) and ArcGIS (version 10.2) (Esri 2013).

## 3 Results

### 3.1 Model Accuracy Evaluation

Based on the evaluation results of each species' individual models, we constructed an ensemble model, in which GBM (ROC = 0.903, TSS = 0.97) achieved the highest average performance and demonstrated relatively high stability across multiple iterations (Figure S2; Table S4). For ensemble models, we tested four different approaches (EMmean, EMmedian, EMca, and EMwmean). Among them, EMca had the highest ROC and TSS values and was therefore selected for further visualization and analysis (Figure S3; Table S5).

### 3.2 Potential Distribution Area of *Pachypodium* Species

The three species with the largest distribution ranges are *P. lealii* (391.13 × 10^3^ km^2^), *P. succulentum* (301.19 × 10^3^ km^2^), and *P. saundersii* (161.29 × 10^3^ km^2^). In contrast, *P. ambongense* holds the smallest distribution range and occupies an area of only 342.792 km^2^ (Figures S4 and S5; Table S1).

**FIGURE 2:**
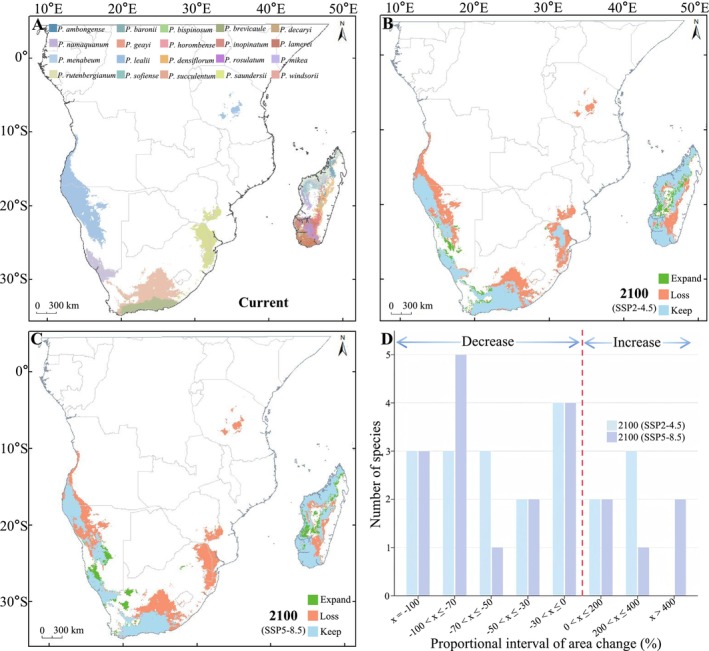
Changes in the potential suitable areas of *Pachypodium* species under climate change. (A) Current distribution range of 20 *Pachypodium* species, with different colors representing different species. (B) Distribution changes by 2100 under the SSP2‐4.5 scenario, relative to current conditions. (C) Distribution changes by 2100 under the SSP5‐8.5 scenario, relative to current conditions. Blue: Areas where the original habitat remains unchanged; green: Newly suitable areas; red: Areas where habitats are lost. (D) The proportion of habitat changes for species under two climate scenarios, where the x‐axis represents habitat change intervals (negative values indicate habitat loss, positive values indicate habitat gain), and the y‐axis represents the number of species.

Relative to current habitats, under the SSP2‐4.5 climate scenario, future habitat areas loss (922.52 × 10^3^ km^2^) is projected to be concentrated in the inland regions of Angola and Namibia, Tanzania, central and northeastern inland South Africa, the border areas between Mozambique and Zimbabwe, as well as parts of central Madagascar. Future habitat areas expansion (307.57 × 10^3^ km^2^) is primarily expected in southwestern Namibia, southwestern South Africa, and along the western coast of Madagascar, extending inland toward central regions. Retained native habitats areas are projected to be predominantly concentrated in areas near coastal zones (Figure 2B). Under the SSP5‐8.5 climate scenario, the range of future habitat areas loss for *Pachypodium* species is expected to expand further (100.99 × 10^3^ km^2^), with habitats in eastern South Africa and Mozambique projected to be completely lost. Newly suitable habitats will primarily emerge in southwestern Namibia and southwestern South Africa, gradually extending inland (Figure 2C). Relative to current habitats, under the SSP2‐4.5 scenario, the proportion of retained native habitats areas for *Pachypodium* species is projected to be 45.8% (778.78 × 10^3^ km^2^), with habitat areas loss reaching 54.2% (922.52 × 10^3^ km^2^) and newly gained suitable habitats areas accounting for 28.3% (307.57 × 10^3^ km^2^) (Figure 2B; Table S6). Under the SSP5‐8.5 scenario, the proportion of retained native habitats areas decreases to 40.6% (691.4 × 10^3^ km^2^), habitat areas loss increases to 59.4% (100.99 × 10^3^ km^2^), and the proportion of newly gained suitable habitats areas rises to 38.2% (427.23 × 10^3^ km^2^) (Table S6).

Fifteen *Pachypodium* species lost their habitat and five increased under the climate scenario. Regarding habitat loss, the number of species remains consistent across both climate scenarios across the loss categories of 100%, 30%–50%, and 0%–30% in proportion. In the range of 100%–70% habitat loss, the number of species under the SSP5‐8.5 climate scenario exceeds that under the SSP2‐4.5 scenario by two species, namely *P. lamerei* and *P. sofiense* , which, under the SSP2‐4.5 scenario, have habitat loss simultaneously in the 70%–50% range. For habitat area increase, the number of species remains consistent when the increase is up to 200%. In the range of 200%–400% increase, the number of species under the SSP2‐4.5 scenario exceeds that under the SSP5‐8.5 scenario by two species, namely *P. decaryi* and *P. windsorii* , which exhibit a habitat area increase of over 400% under the SSP5‐8.5 scenario (Figure 2D; Table S1).

The contribution of environmental factors to the simulation of potential suitable distribution areas varies among species (Figure S6). Annual precipitation (Bio12) is the dominant factor influencing the habitat suitability of *P. geayi* , *P. namaquanum* , *P. horombense* , and *P. mikea* , with contribution rates exceeding 60% for all four species. In comparison, precipitation seasonality (Bio15) is the primary driver for *P. bispinosum* and *P. succulentum* , with contribution rates of 75% and 35.3%, respectively. For *P. lealii* , Bio12 is the dominant factor, contributing 55%, followed by Bio15 at 19.8%. Meanwhile, the habitat suitability of *P. saundersii* is jointly influenced by the minimum temperature of the coldest month (Bio6) and Bio12, with contribution rates of 41.8% and 45.7% (Figure S6).

### 3.3 Reassessment of IUCN Categories for *Pachypodium* Species

**FIGURE 3:**
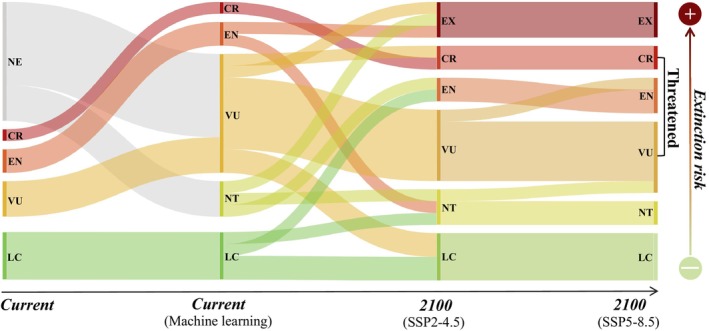
Changes in the IUCN Red List categories of *Pachypodium* species under climate change. Arrows represent the time scale, with lines indicating the Red List status of *Pachypodium* species at Currently, after supplementing with machine learning results, and under two climate scenarios by 2100. The thickness of the lines corresponds to the number of species. CR, critically endangered; EN,endangered; EX, extinct in the wild; NT, near threatened; LC, least concern; NE, not evaluated; VU, vulnerable.

Among the 10 *Pachypodium* species without current IUCN categories, 7 species were classified as VU, and 3 as NT. Under future climate change in the SSP2‐4.5 scenario, the IUCN categories of 10 species remained unchanged, extinction risks increased for 7 species, and decreased for 3 species. Specifically, *P. mikea* , *P. menabeum* , and *P*. *horobinse* were classified as EX. *P*. *brevicaule* was elevated from VU to CR, while *P. saundersii* and *P. geayi* were reclassified from NT and LC, respectively, to EN. *P*. *lamerei* was reclassified from LC to NT. Conversely, *P. baronii* was downgraded from EN to NT, and *P. windsorii* from VU to LC. Compared with the SSP2‐4.5 scenario, under the SSP5‐8.5 climate scenario, extinction risks increased for only two species: *P. sofiense* was elevated from VU to EN, and *P. lamerei* from NT to VU, while other species retained their categories. The number of threatened species under the SSP2‐4.5 scenario is 13, while under the SSP5‐8.5 scenario, it increases to 14 (Figure 3; Table S1).

### 3.4 Conservation Challenges of *Pachypodium* Species

**FIGURE 4:**
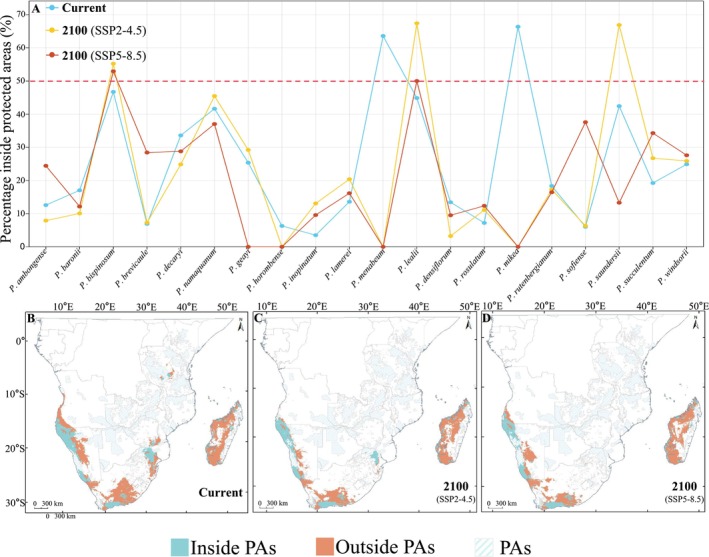
Changes in *Pachypodium* species distribution and protected areas coverage under climate change. (A) The overlap proportion of 20 *Pachypodium* species with protected areas across three time periods: Blue: Current period; yellow: SSP2‐4.5 climate scenario for 2100; red: SSP5‐8.5 climate scenario for 2100. (B) Current relationship between *Pachypodium* and protected areas. (C) Relationship between *Pachypodium* and protected areas under the SSP2‐4.5 scenario for 2100. (D) Relationship between *Pachypodium* and protected areas under the SSP5‐8.5 scenario for 2100.

Under the current climate scenario, only *P. mikea* and *P. menabeum* have more than 50% of their habitats located within protected areas. However, both species are projected to lose all suitable habitats under future climate change. In the SSP2‐4.5 scenario, the species with over 50% of their habitats within protected areas are *P. bispinosum* , *P. lealii* , and *P. saundersii* , while in the SSP5‐8.5 scenario, only *P. bispinosum* and *P. lealii* remain above this threshold. Compared with current protected habitat coverage, *P. brevicaule* , *P. rutenbergianum* , *P. sofiense* , and *P. windsorii* show no significant changes in the SSP2‐4.5 scenario. However, the habitat protection for *P. ambongense* , *P. baronii* , *P. decaryi* , and *P. densiflorum* is projected to decrease further. Under the SSP5‐8.5 scenario, only nine species experience increased habitat protection: *P. ambongense* , *P. bispinosum* , *P. brevicaule* , *P. inopinatum* , *P. lamerei* , *P. lealii* , *P. rosulatum* , *P. succulentum* , and *P. windsorii* . While for the remaining species, the proportion of their habitats within protected areas is expected to decline (Figure 4A; Table S1).

The total area of protected habitats for the *Pachypodium* species shows a clear decreasing trend from its current status to 2100. The current protected area for this genus is 468.511 × 10^3^ km^2^. Under the SSP2‐4.5 and SSP5‐8.5 climate scenarios, the extent of protected habitats by the year 2100 is projected to decrease to 356.512 × 10^3^ km^2^ and 326.145 × 10^3^ km^2^, respectively. Compared with the current protected area, the habitat area under protection in 2100 is reduced by 23.9% in the SSP2‐4.5 scenario and further declines by 30.39% under SSP5‐8.5. Spatially, unprotected *Pachypodium* habitats are primarily concentrated in central Madagascar, the western coastal regions of Madagascar, southwestern South Africa, and inland areas of Namibia (Figure 4B–D; Table S1).

## 4 Discussion

Climate change is reshaping global ecosystems and biodiversity, leading to alterations in species distributions, interactions, and population dynamics (Pecl et al. 2017; Urban 2024). The effects of climate change on plant habitats are typically manifested as a reduction in climatically suitable areas, as well as shifts in habitat ranges (Dang et al. 2021; Van Beest et al. 2023). Our findings indicate that 15 *Pachypodium* species are expected to experience potential habitat loss, while the habitat areas of five species are projected to expand under future climate scenarios (SSP5‐8.5 and SSP2‐4.5) (Figures S3 and S4; Table S1), demonstrating distinct climatic adaptive responses among different species to climate change.

Among the influence of various environmental factors, Bio12 and Bio15 are key drivers regulating the potential distribution of *Pachypodium* species (e.g., *P. bispinosum* , *P. horombense* , *P. inopinatum* , *P. lealii* , and *P. succulentum* ) (Figure S6). Based on the phylogenetic framework constructed by Burge et al. (2013), we found that *P. bispinosum* and *P. succulentum* , which are primarily influenced by Bio15, form a monophyletic group on the phylogenetic tree, and their potential suitable areas are concentrated in the narrow coastal region of southern South Africa. Similarly, *P. lamerei* , *P. mikea* , and *P. geayi* , regulated by Bio12, are sister species with highly overlapping suitable areas along the western coastal zone of Madagascar (Figures S4–S6). This pattern suggests that closely related species may exhibit a coordinated response to specific climatic factors (e.g., Bio12 or Bio15) due to shared ancestral ecological niche conservatism (phylogenetic niche conservatism), thereby facing similar spatial distribution constraints under future climate change (Martínez de León and Moreno‐Letelier 2025; Qian et al. 2022).

The impact of climate change on the distribution patterns of *Pachypodium* species reveals that coastal‐dwelling species, such as *P. rutenbergianum* , *P. decaryi* , *P. windsorii* , and *P. baronii* , may benefit from climate change. Their current habitat areas are expected to expand significantly (Table S1), with future potential distributions showing a tendency to shift toward coastal regions (Figure S4). This spatial expansion is likely driven by favorable conditions, such as increased precipitation in coastal areas under climate change (Beaudin et al. 2023). Similar patterns have been observed in other climate‐sensitive plant groups, including the genus *Abies* and the *Xerophyta*, whose populations have demonstrated adaptive responses by migrating to more suitable habitats (Tekin et al. 2022; Wanga et al. 2024). However, the spatial heterogeneity of climate change poses challenges for some species to achieve adaptive migration. For example, the endemic Malagasy species *P. ambongense* and *P. brevicaule* , characterized by their narrow distribution ranges and strict ecological niche requirements, face significant geographical dispersal limitations and ecological barriers, substantially increasing their risk of extinction.

The application of deep learning architectures and integrated ensemble modeling frameworks has significantly enhanced the predictive accuracy in extinction risks evaluation for *Pachypodium* species. Our results reveal that IUCN underestimates the impacts of climate change on extinction risks for *Pachypodium* species. For example, *P. mikea* , currently classified as EN by IUCN, is projected to lose its entire habitat under the SSP5‐8.5 scenario, warranting reclassification to EX. Similarly, the IUCN status of *P. brevicaule* escalates from VU to CR. For the unevaluated *P. saundersii* , our criteria classify it as EN, establishing a novel assessment framework for climate‐vulnerable species lacking formal IUCN evaluation. These findings further demonstrate that climate change significantly increases the complexity of species extinction risks by influencing habitat shifts and distribution changes (Lambers 2015). Machine learning technology provides a novel approach to address this challenge (Jordan and Mitchell 2015; Taye 2023). By accurately simulating future habitat changes and assessing extinction risks, it offers valuable scientific evidence and data support for global species conservation and updates to the IUCN Red List (Huang et al. 2023). Moving forward, integrating the impacts of climate change with machine learning‐based extinction risk assessments and regularly updating these evaluations will be a critical step in global biodiversity conservation efforts (Eyring et al. 2024).

Climate change is driving habitat shifts for many *Pachypodium* species, with some, like *P. geayi* and *P. saundersii* , expanding beyond protected areas (Figure 4; Table S1). Without adequate protection measures, these species may face severe habitat pressures, significantly increasing their risk of extinction. In contrast, for species such as *P. sofiense* and *P. succulentum* , our findings suggest that their habitats may shift into existing protected areas under climate change, potentially enhancing their conservation (Figure 4; Table S1). However, such cases are exceptions rather than the rule. The majority of *Pachypodium* species are still at risk of habitat loss, highlighting the lack of flexibility and adaptability in the current protected area network to address the challenges of biodiversity conservation in the context of climate change (Hoveka et al. 2022).

The intensification of climate change has profound impacts on plant habitats that extend well beyond the scope of current protected area planning and management. While existing protected areas have played a crucial role in mitigating anthropogenic threats to species, they are increasingly revealing their limitations in the face of climate change (Hoveka et al. 2022; Peng et al. 2023). Firstly, the static geographic boundaries of many protected areas are insufficient to accommodate the dynamic shifts in species habitats driven by climate change, as these habitat changes often extend beyond existing protected zones (Hoveka et al. 2022; Wu et al. 2023). Moreover, the establishment and management of protected areas have traditionally overlooked the influence of climate change on species migration pathways, resulting in inadequate support for habitat shifts and expansions within protected areas (Mi et al. 2023; Peng et al. 2021). Simultaneously, climate change‐induced extreme weather events, such as droughts and heavy rainfall, can directly disrupt ecological balance within protected areas, leading to habitat degradation and reduced habitat quality (Ainsworth et al. 2020; Spinoni et al. 2021). This challenge is particularly pronounced in tropical and subtropical regions, where the frequency and intensity of extreme weather events are expected to increase, further threatening habitat stability and species persistence.

Amid the intensifying impacts of climate change, the current protected area system requires reform and adaptive adjustments to address the challenges posed by future habitat shifts and habitat loss for species. Expanding the coverage of protected areas, establishing ecological corridors, enhancing the management flexibility of protected zones, and integrating strategies such as in situ, near‐situ, and ex situ conservation (Cogoni et al. 2021; Mestanza‐Ramón et al. 2020; Popescu et al. 2022; Yang et al. 2020). Concurrently, population recovery initiatives for endangered species should be implemented through integrated conservation strategies encompassing artificial propagation (including tissue culture technology), seed banking systems, and scientifically guided reintroduction programs. Recent studies have demonstrated the efficacy of these methodologies in enhancing species survival rates and ecosystem restoration (Hoyle et al. 2023; Kulak et al. 2022; Shao et al. 2022; Ullah et al. 2024). To ensure long‐term species viability, it is imperative to establish concurrent genetic diversity monitoring and preservation protocols during conservation implementation (Wang et al. 2020). In addition, strict enforcement of laws and regulations is necessary, with increased penalties for illegal harvesting and trade (Challender et al. 2023). The comprehensive implementation of these measures will provide systematic solutions for biodiversity conservation under climate change, ensuring the long‐term survival of endangered species and the sustained functioning of ecological processes.

## 5 Conclusion

This study employs species distribution models to predict changes in the distribution patterns of 20 *Pachypodium* species under climate change scenarios. Additionally, machine learning methods are applied to assess the extinction risk of these species. The study reveals the severe extinction risks for these species and highlights the inadequacies of the current protected area system in addressing climate change impacts. The results show that under future climate change, the suitable habitats for many *Pachypodium* species will significantly shrink, and some species, such as *P. mikea* , *P. horombense* , and *P. menabeum* , will completely lose their suitable habitats, facing extinction threats. Consequently, the habitats of certain species, such as *P. baronii* , *P. decaryi* , *P. rutenbergianum* , and *P. windsorii* , are projected to expand in specific regions, indicating that different species have varying levels of adaptability and that climate change will affect species differently. Future IUCN categories suggest that the threat levels for most species will increase, particularly under high‐emission scenarios. Furthermore, the conservation gap for several species will significantly increase in the future. To address these challenges, future conservation strategies must integrate flexible protected area management, habitat restoration, and adaptive measures such as genetic diversity monitoring and artificial propagation to ensure the long‐term survival of *Pachypodium* species.

## Author Contributions

**Yu Chen:** methodology (equal), resources (equal), software (equal), visualization (equal), writing – original draft (equal). **Qisong Wan:** data curation (equal), formal analysis (equal), investigation (equal), methodology (equal), visualization (equal). **Shenglan Du:** writing – original draft (equal), writing – review and editing (equal). **Hillary Otieno Otieno:** methodology (equal), writing – review and editing (equal). **Haingotiana Johary Andrianjatovo:** investigation (equal), writing – review and editing (equal). **Maxwell Njoroge Njenga:** investigation (equal), writing – review and editing (equal). **Yuvenalis Morara Mbuni:** investigation (equal), resources (equal). **Neng Wei:** resources (equal), supervision (equal). **Jitao Li:** project administration (equal), supervision (equal). **Shengwei Wang:** methodology (equal), project administration (equal), supervision (equal), validation (equal), visualization (equal), writing – review and editing (equal).

## Ethics Statement

The authors have nothing to report.

## Conflicts of Interest

The authors declare no conflicts of interest.

## Supporting information


**Data S1:** ece371926‐sup‐0001‐Supinfo.pdf.

## Data Availability

The species distribution data for this study were mainly obtained from the Global Biodiversity Information Facility (GBIF, https://www.gbif.org), RAINBIO mega database (Rainbio, http://rainbio.cesab.org), and Tsimbazaza Botanical and Zoological Park Herbarium (TAN). The digitized specimen data can be requested from the corresponding author. The bioclimatic variables obtained from WorldClim (http://www.worldclim.org); elevation data were sourced from the GMRT database (www.gmrt.org).
