# Supplementary material for: Extinction Risk Assessment and Conservation of the Pachypodium Under Climate Change
Source: Ecol Evol. 2025 Aug 8;15(8):e71926. doi: 10.1002/ece3.71926 (PMC12334552; doi:10.1002/ece3.71926)
Supplement: Supplementary file 1 — Data S1: ece371926‐sup‐0001‐Supinfo.pdf. [file ECE3-15-e71926-s001.pdf]

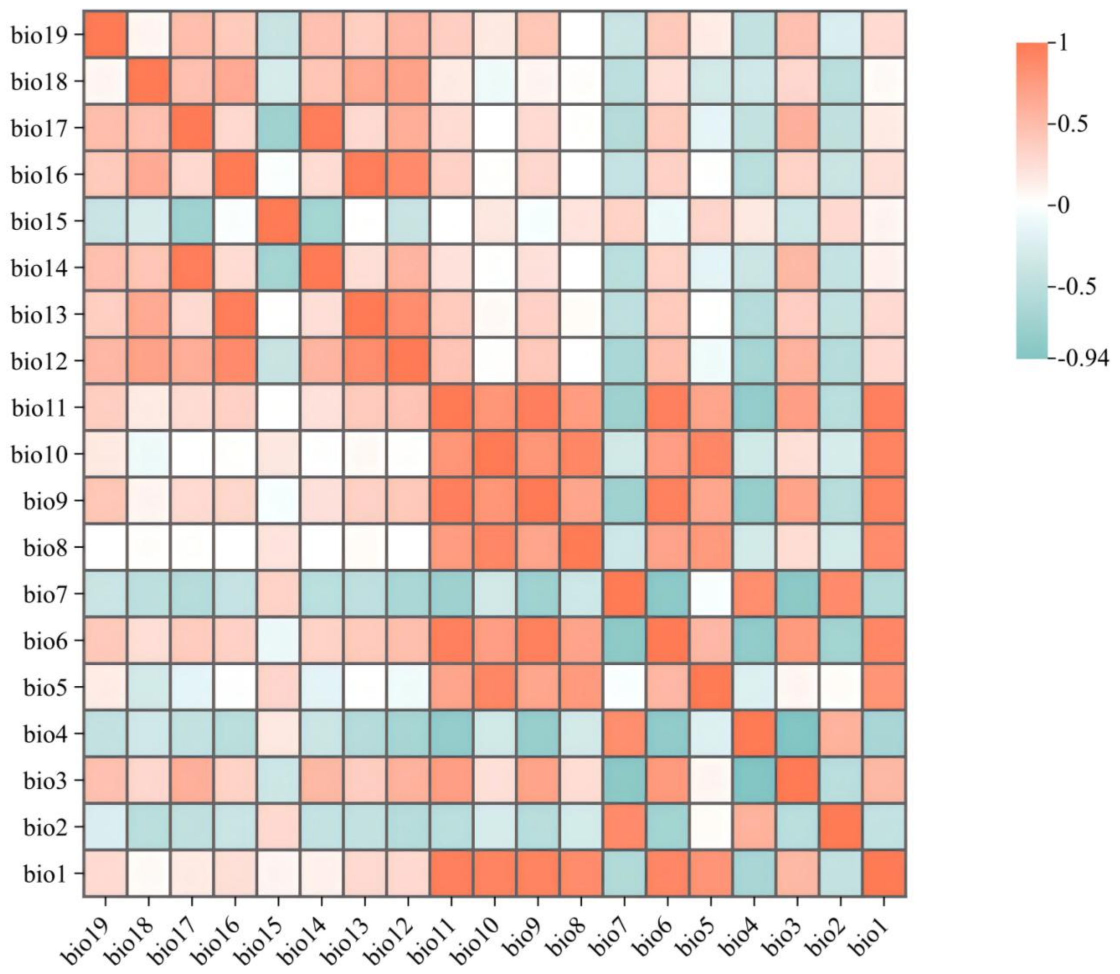

Fig. S1 | Pearson correlation analysis results for climate variables. **Note:** Variables with correlation coefficients greater than 0.75 were excluded.

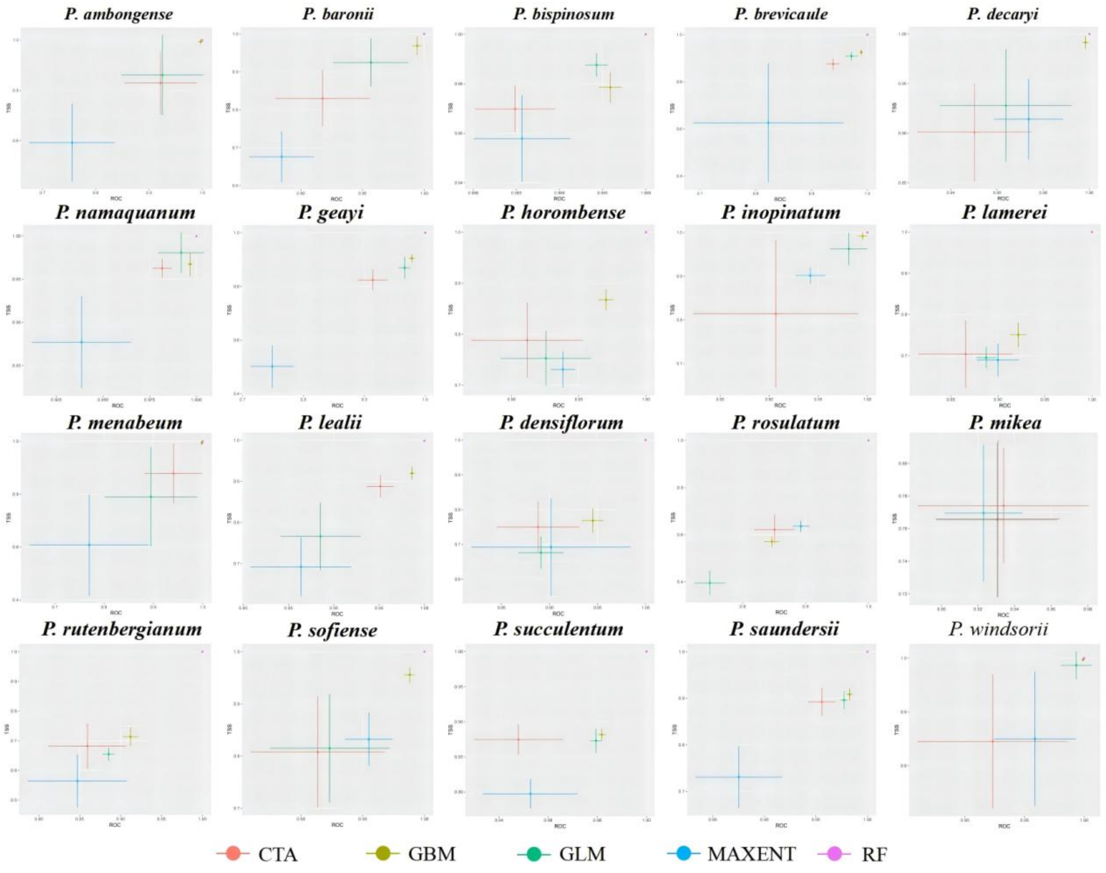

Fig. S2 | Evaluation results of single models for 20 *Pachypodium* species. **CTA:** Classification Tree Analysis; **GBM:** Generalized Boosted Model; **GLM:** Generalized Linear Model; **MAXENT:** Maximum Entropy; **RF:** Random Forest.

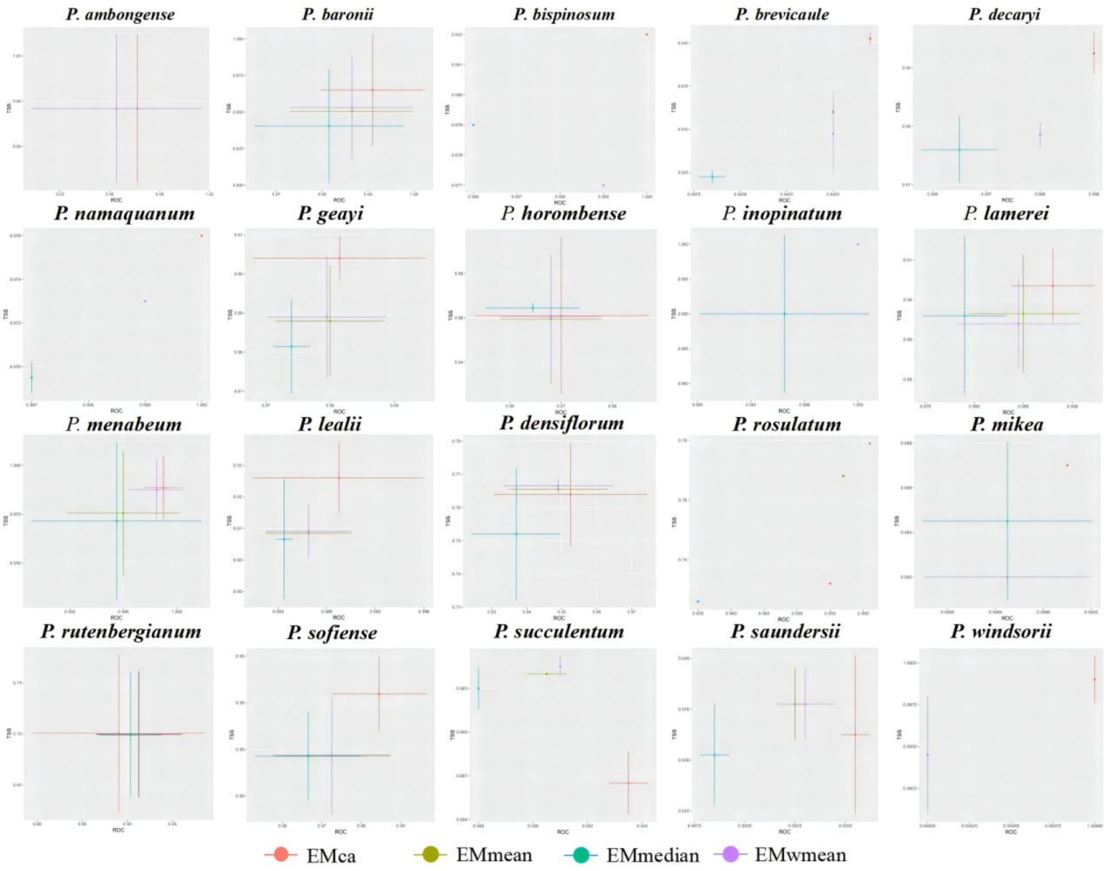

Fig. S3 | Evaluation results of ensemble models for 20 *Pachypodium* species. **EMca:** Classification Accuracy; **EMmean:** Mean Value; **EMmedian:** Median Value; **EMwmean:** Weighted Mean.

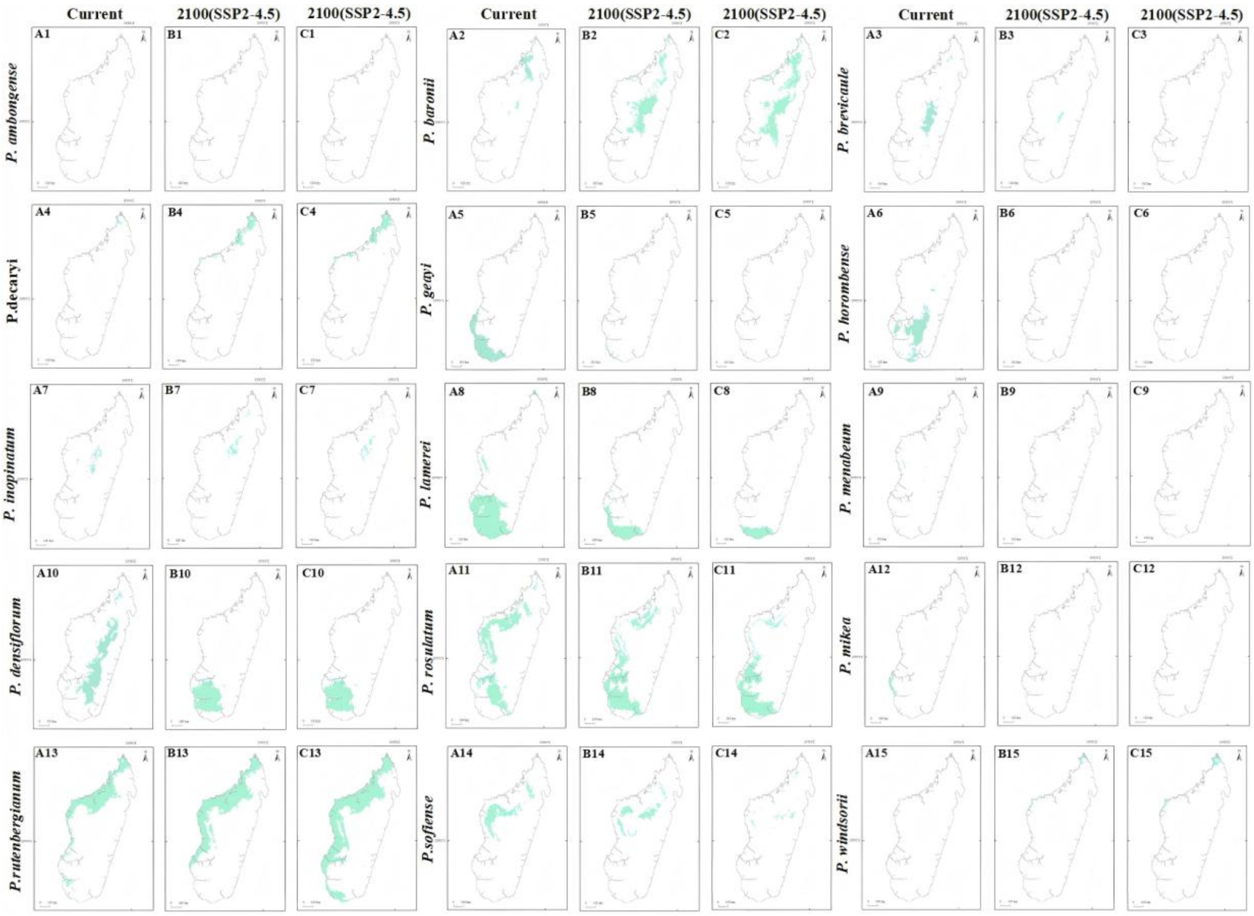

Fig. S4 | Potential Habitats of 15 *Pachypodium* Species in Madagascar Across Three Time Periods. **A:** Current; **B:** 2100(SSP2-4.5); **C:** 2100(SSP5-8.5).

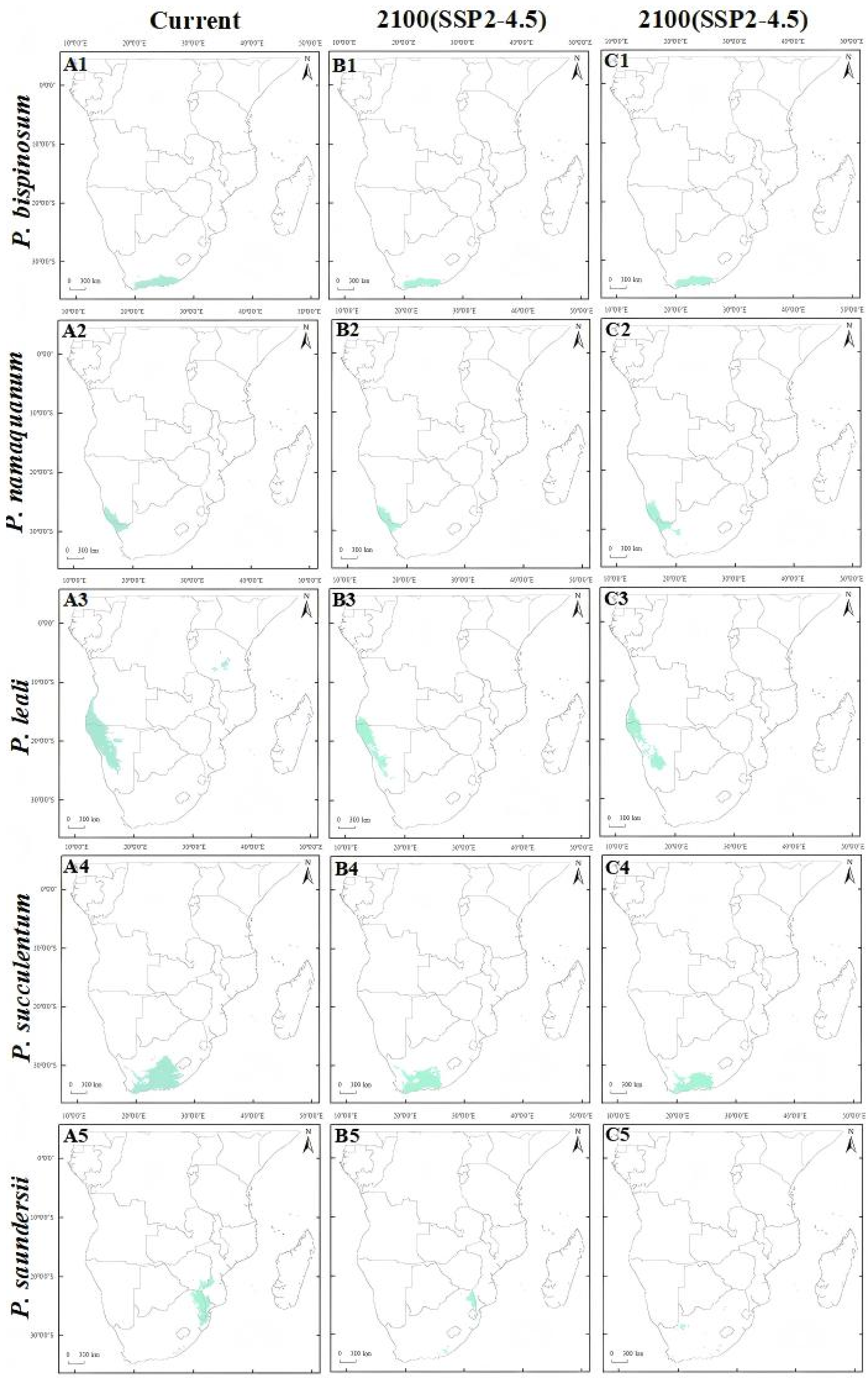

Fig. S5 | Potential Habitats of 5 *Pachypodium* Species in on the African continent Across Three Time Periods. **A**: Current; **B**: 2100(SSP2-4.5); **C**: 2100(SSP5-8.5).

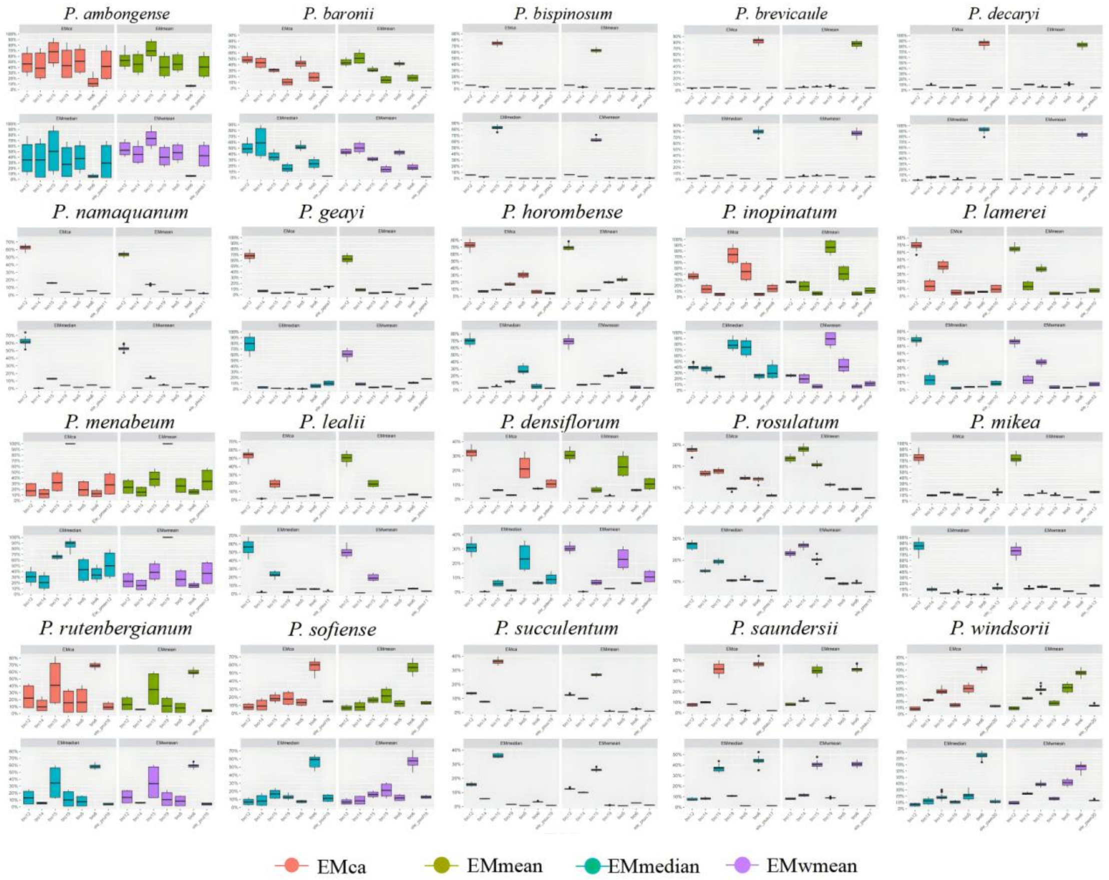

Fig. S6 | Contribution rates of environmental variables in ensemble models for 20 *Pachypodium* species.

ble S1. Changes in IUCN Categories for 20 *Pachypodium* species after machine learning analysis and reassessment under climate change scenarios. **IUCN Category:** IUCN Categories results in: IUCN Categories results in three time periods (current, 2100 under SSP2-4.5 and SSP5-8.5 climate scenarios); **Machine Learning**：Preliminary assessment results through machine learning; **Potential Suitable Area:** Habitat suitability area (km²) for *Pachypodium* species across three time periods; **Inside of PAs**： Proportion (%) of potential suitable habitat for *Pachypodium* species inside existing protected areas (PAs) across three time periods. **EX:** Extinct in the Wild; **CR:** Critically Endangered; **EN:** Endangered; VU: Vulnerable; **NT**: Near Threatened; **LC:** Least Concern; **NE:** Not Evaluated.

Family	Genus	Species Name	IUCN Categories				Potential Suitable Area（km²）			Inside of PAs（%）		
			Current	Machine Learning	2100（SSP2-4.5）	2100（SSP5-8.5）	Current	2100（SSP2-4.5）	2100（SSP5-8.5）	Current	2100（SSP2-4.5）	2100（SSP5-8.5）
Apocynaceae	Pachypodium	*P. ambongense*	NE	VU	VU	VU	342.792	175.439	153.976	12.577	7.90	24.43
Apocynaceae	Pachypodium	*P. baronii*	EN	EN	NT	NT	21605.28	68296.418	91687.248	17.034	10.06	12.16
Apocynaceae	Pachypodium	*P. bispinosum*	NE	NT	NT	NT	94277.89	74531.618	79607.647	46.723	55.227	52.93
Apocynaceae	Pachypodium	*P. brevicaule*	VU	VU	CR	CR	28529.94	2726.530	193.784	6.925	7.283	28.43
Apocynaceae	Pachypodium	*P. decaryi*	NE	VU	LC	LC	5075.555	23807.199	31809.308	33.596	24.85	28.79
Apocynaceae	Pachypodium	*P. namaquanum*	LC	LC	LC	LC	75922.14	75813.422	111431.82	41.643	45.46	37.03
Apocynaceae	Pachypodium	*P. geayi*	LC	LC	EN	EN	52823.69	3344.038	340.260	25.366	29.23	0.001
Apocynaceae	Pachypodium	*P. .horombense*	NE	NT	EX	EX	57071.31	0	93.701	6.299	0	0
Apocynaceae	Pachypodium	*P. inopinatum*	CR	CR	CR	CR	10858.88	9446.165	7771.202	3.505	13.08	9.58
Apocynaceae	Pachypodium	*P. lamerei*	LC	LC	NT	VU	121846.9	42488.118	24987.922	13.586	20.08	16.16
Apocynaceae	Pachypodium	*P. menabeum*	NE	VU	EX	EX	1285.737	0	36.415	63.585	0	0

Apocynaceae	Pachypodium	*P. lealii*	VU	VU	VU	VU	391134.6	189878.76	209055.84	44.864	67.40	50.003
Apocynaceae	Pachypodium	*P. densiflorum*	NE	VU	VU	VU	93564.15	36472.720	67671.951	13.435	3.24	9.51
Apocynaceae	Pachypodium	*P. rosulatum*	NE	VU	VU	VU	118025.1	120433.48	103135.61	7.20	11.12	12.36
Apocynaceae	Pachypodium	*P. mikea*	EN	EN	EX	EX	5741.221	0	33.624	66.376	0	0
Apocynaceae	Pachypodium	*P. rutenbergianum*	LC	LC	LC	LC	111967.4	153576.37	197259.771	18.352	17.26	16.45
Apocynaceae	Pachypodium	*P. sofiense*	VU	VU	VU	EN	46185.48	33363.583	7065.2160	6.041	6.31	37.58
Apocynaceae	Pachypodium	*P. succulentum*	NE	VU	VU	VU	301191.4	197548.22	151474.02	19.231	26.72	34.29
Apocynaceae	Pachypodium	*P. saundersii*	NE	NT	EN	EN	161293.3	46246.467	20106.263	42.434	66.90	13.29
Apocynaceae	Pachypodium	*P. windsorii*	NE	VU	LC	LC	2559.990	8205.091	14877.017	24.895	25.90	27.60

Table S2. Environmental variables used in this study. The asterisk (*) represents the factors used in the species distribution model in this study.

Abbreviation	Environmental Variables	Units
Bio1	Annual Mean Temperature	℃
Bio2	Mean Diurnal Range	℃
Bio3	Isothermality	-
Bio4	Temperature Seasonality	-
Bio5 *	Max Temperature of Warmest Month	℃
Bio6 *	Min Temperature of Coldest Month	℃
Bio7	Temperature Annual Range	℃
Bio8	Mean Temperature of Wettest Quarter	℃
Bio9	Mean Temperature of Driest Quarter	℃
Bio10	Mean Temperature of Warmest Quarter	℃
Bio11	Mean Temperature of Coldest Quarter	℃
Bio12*	Annual Precipitation	mm
Bio13	Precipitation of Wettest Month	mm
Bio14*	Precipitation of Driest Month	mm
Bio15 *	Precipitation Seasonality	-
Bio16	Precipitation of Wettest Quarter	mm
Bio17	Precipitation of Driest Quarter	mm
Bio18	Precipitation of Warmest Quarter	mm
Bio19 *	Precipitation of Coldest Quarter	mm
Elev *	Elevation	m

Table S3. Reassessment of IUCN Categories for *Pachypodium* Species Under Future Climate Scenarios. **Preliminary Assessment**: IUCN categories of 20 *Pachypodium* species, with missing classifications supplemented using machine learning; **Proportion of Habitat Loss/Expand**: Proportion (%) of habitat area change (loss/expansion) under climate scenarios. **EX:** Extinct in the Wild; **CR:** Critically Endangered; **EN:** Endangered; VU: Vulnerable; **NT**: Near Threatened; **LC:** Least Concern; **NE:** Not Evaluated.

Preliminary Assessment	Proportion of Habitat Loss/Expand (%)								
	Loss					Expand			
	100%	90%	70%	50%	30%	50%	100%	150%	200%
CR	EX	EX	CR	CR	CR	CR	CR	EN	VU
EN	EX	CR	EN	EN	EN	EN	EN	VU	NT
VU	EX	CR	EN	VU	VU	VU	VU	NT	LC
NT	EX	CR	EN	VU	NT	NT	NT	LC	LC
LC	EX	EN	VU	NT	LC	LC	LC	LC	LC

Table S4. Contribution of 20 *Pachypodium* species under five single models. **CTA:** Classification Tree Analysis; **GBM:** Generalized Boosted Model; **GLM:** Generalized Linear Model; **MAXENT:** Maximum Entropy; **RF:** Random Forest. **ROC**:Receiver Operating Characteristic Curve; **TSS**:True Skill Statistics.

**Species Name**	**Single-model contribution rate**									
	**CTA**		**GBM**		**GLM**		**MAXENT**		**RF**	
	**ROC**	**TSS**	**ROC**	**TSS**	**ROC**	**TSS**	**ROC**	**TSS**	**ROC**	**TSS**
P. ambongense	0.81	0.9	0.99	0.99	0.83	0.925	0.59	0.755	1	1
P. baronii	0.835	0.92	0.97	0.97	0.925	0.96	0.675	0.87	1	1
P. bispinosum	0.97	0.9845	0.979	0.996	0.987	0.994	0.958	0.986	1	1
P. brevicaule	0.875	0.94	0.92	0.99	0.91	0.975	0.63	0.823	1	1
P. decaryi	0.9	0.95	0.99	0.996	0.928	0.964	0.9135	0.974	1	1
P. namaquanum	0.962	0.979	0.966	0.992	0.977	0.985	0.876	0.938	1	1
P. geayi	0.835	0.92	0.908	0.972	0.87	0.965	0.505	0.75	1	1
P. .horombense	0.788	0.912	0.875	0.97	0.758	0.926	0.73	0.937	1	1
P. inopinatum	0.81	0.91	0.99	0.987	0.96	0.979	0.941	0.939	1	1
P. lamerei	0.71	0.866	0.75	0.922	0.695	0.888	0.68	0.9	1	1
P. menabeum	0.88	0.94	0.99	0.99	0.792	0.892	0.608	0.77	1	1
P. lealii	0.88	0.952	0.922	0.975	0.763	0.87	0.695	0.862	1	1
P. densiflorum	0.75	0.875	0.77	0.942	0.675	0.88	0.695	0.912	1	1
P. rosulatum	0.612	0.85	0.582	0.842	0.4	0.748	0.634	0.88	1	1
P. mikea	0.984	0.992	0.998	0.998	0.954	0.9762	0.981	0.9924	1	1
P.rutenbergianum	0.688	0.86	0.718	0.913	0.652	0.881	0.62	0.849	1	1
P. sofiense	0.808	0.913	0.96	0.988	0.82	0.924	0.832	0.957	1	1
P. succulentum	0.875	0.947	0.879	0.982	0.874	0.979	0.793	0.953	1	1
P. saundersii	0.89	0.957	0.913	0.98	0.898	0.976	0.78	0.875	1	1
P. windsorii	0.694	0.923	0.999	0.999	0.985	0.99	0.7	0.959	1	1
SUM	16.56	18.51	18.07	19.39	16.65	18.68	14.84	17.88	20	20
MEAN	0.828	0.925	0.904	0.970	0.833	0.934	0.742	0.894	1	1

Table S5. Contribution of 20 *Pachypodium* species under ensemble models. **Abbreviations:**EMmean: Mean Value; EMmedian: Median Value; EMca： Classification Accuracy; EMwmean: Weighted Mean. **ROC**:Receiver Operating Characteristic Curve; **TSS**:True Skill Statistic.

**Species Name**	**Ensemble-model contribution rate**							
	**EMmean**		**EMmedian**		**EMca**		**EMwmean**	
	**ROC**	**TSS**	**ROC**	**TSS**	**ROC**	**TSS**	**ROC**	**TSS**
P. ambongense	0	0	0	0	0.977	0.954	0.9635	0.954
P. baronii	0.9867	0.9504	0.9818	0.941	0.9915	0.9646	0.9867	0.9536
P. bispinosum	0	0	0.996	0.979	1	0.982	0.999	0.977
P. brevicaule	0.995	0.932	0.9885	0.9242	0.969	0.9407	0.995	0.9295
P. decaryi	0	0	0.9965	0.9761	0.999	0.9926	0.998	0.972
P. namaquanum	0	0	0.997	0.9695	1	0.976	0.999	0.973
P. geayi	0.98	0.8876	0.974	0.8824	0.9823	0.9042	0.9796	0.8891
P. .horombense	0.9676	0.8599	0.9648	0.8644	0.97	0.861	0.9676	0.8602
P. inopinatum	0	0	0.9949	0.99	0	0	1	1
P. lamerei	0.985	0.8663	0.9785	0.8959	0.9879	0.9038	0.9845	0.8942
P. menabeum	0.996	0.9751	0.9954	0.9713	0.999	0.988	0.9986	0.9875
P. lealii	0.9864	0.909	0.9847	0.9068	0.9895	0.9256	0.9864	0.9091
P. densiflorum	0.9492	0.7651	0.9372	0.7523	0.9527	0.7643	0.9492	0.7662
P. rosulatum	0.9572	0.7684	0.935	0.7246	0.955	0.732	0.9634	0.779
P. mikea	0	0	0.9985	0.9945	0.999	0.997	0.9985	0.992
P. rutenbergianum	0.913	0.6997	0.904	0.6995	0.889	0.70	0.913	0.6998
P. sofiense	0.9725	0.8425	0.9765	0.8424	0.9849	0.9105	0.9725	0.843
P. succulentum	0.9905	0.9041	0.988	0.903	0.9935	0.8964	0.991	0.9045
P. saundersii	0.9925	0.9357	0.9886	0.9307	0.9325	0.9968	0.9931	0.9357
P. windsorii	0	0	0	0	1	0.999	0.999	0.9944
SUM	12.672	11.296	17.58	16.148	18.572	17.389	19.638	18.214
MEAN	0.9747	0.8689	0.9767	0.8971	0.9775	0.9152	0.9819	0.9107

Table S6. Habitat and area changes across *Pachypodium* species under climate scenarios. **Periods:** Habitat area of *Pachypodium* species in three time periods (current, 2100 SSP2-4.5, and SSP5-8.5 climate scenarios; **Keep**: Habitat area remained constant under both periods; **Loss**: Loss of habitat area compared to current; **Expand**: Increased compared to current habitat area.

**Periods**	**Current**		**2100（SSP2-4.5）**		**2100（SSP5-8.5）**	
**Area**	1701302.85		1086353.63		1118628.86	
-	Area (km^2)	Proportion (%)	Area (km^2)	Proportion (%)	Area (km^2)	Proportion (%)
**Keep**	-	-	778780.03	45.8	691398.87	40.6
**Loss**	-	-	922522.82	54.2	1009903.97	59.4
**Expand**	-	-	307573.60	28.3	427229.98	38.2
